# Generalization of sustained neurophysiological effects of short‐term auditory 13‐Hz stimulation to neighbouring frequency representation in humans

**DOI:** 10.1111/ejn.15513

**Published:** 2021-12-16

**Authors:** Daria F. Kleeva, Anna B. Rebreikina, Gurgen A. Soghoyan, Daria G. Kostanian, Anastacia N. Neklyudova, Olga V. Sysoeva

**Affiliations:** ^1^ Center for Cognitive Research Sirius University of Science and Technology Sochi Russia; ^2^ Center for Bioelectric Interfaces National Research University “Higher School of Economics” Moscow Russia; ^3^ Laboratory of Human Higher Nervous Activity Institute of Higher Nervous Activity and Neurophysiology of RAS Moscow Russia; ^4^ V. Zelman Center for Neurobiology and Brain Restoration Skolkovo Institute of Science and Technology Moscow Russia

**Keywords:** auditory event‐related potential (ERP), electroencephalography (EEG), LTP‐like stimulation/tetanization, perceptual learning

## Abstract

A fuller understanding of the effects of auditory tetanization in humans would inform better language and sensory learning paradigms; however, there are still unanswered questions. Here, we probe sustained changes in the event‐related potentials (ERPs) to 1020‐ and 980‐Hz tones following a rapid presentation of 1020‐Hz tone (every 75 ms, 13.3 Hz, tetanization). Consistent with some previous studies, we revealed the increase in the P2 ERP component after tetanization. Contrary to some other studies, we did not observe the expected N1 increase after tetanization even in the identical experimental sequence. We detected a significant N1 decrease after tetanization. Expanding previous research, we showed that P2 increase and N1 decrease are not specific to the stimulus type (tetanized 1020 Hz and non‐tetanized 980 Hz), suggesting the generalizability of tetanization effect to the not‐stimulated auditory tones, at least to those of the neighbouring frequency. The ERPs' tetanization effects were observed for at least 30 min—the most prolonged interval examined, consistent with the duration of long‐term potentiation, LTP. In addition, the tetanization effects were detectable in the blocks where the participants watched muted videos, an experimental setting that can be easily used in children and other challenging groups. Thus, auditory 13‐Hz stimulation affects brain processing of tones including those of neighbouring frequencies.

## Graphical abstract

A fuller understanding of the effects of auditory tetanization in humans would inform better language and sensory learning paradigms; however, there are still unanswered questions. Here, we probed sustained changes in the event‐related potentials (ERPs) to 1020‐ and 980‐Hz tones following a rapid presentation of 1020‐Hz tone (every 75 ms, 13.3 Hz, tetanization) and found a significant decrease in N1 and increase in P2 amplitude after tetanization for both tetanized and non‐tetanized tone. Thus, auditory 13‐Hz stimulation affects brain processing of tones including those of neighbouring frequencies.

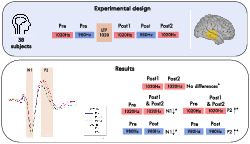

**Abbreviations**
ANOVA: analysis of variances
EEG: electroencephalography
ERPs: event‐related potentials
LTP: long‐term potentiation
MMN: mismatch negativity

## 1 INTRODUCTION

The mechanisms of learning are widely studied in various disciplines—biochemistry, neurophysiology, psychology, and social sciences. The integration of the progress from each discipline can potentially open the prospects for a more in‐depth assessment of learning deficits or their modulation factors. The attempt of such an integration includes the studies of long‐term potentiation (LTP) in the framework of non‐invasive neuroimaging. LTP is a cellular phenomenon and involves the strengthening of synaptic transfer between two neurons, which persists for a long time after the stimulation of a synaptic pathway lasting from several hours to months depending on the stimulation protocol or the brain areas involved (Abraham & Williams, 2003). LTP is considered a crucial mechanism that underlies the activity‐dependent plasticity of the cortex (Takeuchi et al., 2013).

Originally, LTP has been studied at the cellular level in animals as provoked by electrical stimulation (Cooke & Bliss, 2006; Frey & Morris, 1997; Moser et al., 1998); however recently, several authors have shown LTP‐like effects triggered by non‐invasive high‐frequency sensory stimulation/tetanization both in animals (Burgdorf et al., 2019; Clapp, Eckert, et al., 2006; Cooke & Bear, 2012) and humans (Çavuş et al., 2012; Clapp et al., 2012; Kompus & Westerhausen, 2018; Mears & Spencer, 2012; Normann et al., 2007; Teyler et al., 2005). This sensory stimulation is hypothesized to elicit the rhythmic neuronal bursts in the cortex utilizing mechanisms that are involved in the effect of electrical tetanization at cellular level (Kirk et al., 2010; Sanders et al., 2018). The resulting potentiation can be measured non‐invasively using electroencephalography (EEG) as a change of event‐related potentials (ERP)—the summed neuronal responses evoked by the sensory stimulus, contrasting the conditions before and after the stimulation. Moreover, this sensory tetanization has been shown to increase discrimination ability and long‐term memory performance, thus suggesting its link to behavioural improvement in humans (Beste et al., 2011; Clapp et al., 2012; Spriggs et al., 2018).

Most studies apply rapid visual stimulation (e.g., with a frequency of 9 Hz) to induce LTP‐like effects in humans. Such visual tetanization leads to a decrease of the detection thresholds and the reaction time (Beste et al., 2011; Clapp et al., 2012) and is accompanied by an increase in the early component of visual ERP, N1b (Clapp, Muthukumaraswamy, et al., 2006; McNair et al., 2006; Ross et al., 2008; Teyler et al., 2005), as well as the later one, P2 (Spriggs et al., 2018). LTP‐like learning has also been studied in the auditory domain in humans, but the results of these studies are rather inconsistent. The initial study has reported the sustained increase of fronto‐central N1 component in response to 1000‐Hz tone after its presentation with a frequency of 13 Hz for 2 min (Clapp et al., 2005). The same experimental design has been used in three more studies (Lei et al., 2017; Rygvold et al., 2021; Teo et al., 2014), although with rather inconsistent results as the N1 increase has been replicated only in one study but at the different posterior region (Lei et al., 2017), while other studies have found a significant increase of N1‐P2 peak‐to‐peak or P2 amplitude (Rygvold et al., 2021; Teo et al., 2014). Thus, the ERP effects of auditory tetanization need additional examination.

In human studies with visual tetanization (McNair et al., 2006; Ross et al., 2008), LTP effect has been input specific (do not transfer to stimuli of different orientation and spatial frequency). In the auditory domain, stimulus‐specific plasticity has been examined by two research groups using a slightly modified auditory tetanization paradigm compared with what has been described in the previous paragraph. Mears and Spencer (2012) asked participants to respond to 400‐Hz target stimuli interspersed with either 1000‐ or 1500‐Hz standards. The tetanization included only 1000 Hz, which induced the negative shift in ERPs at right temporal channels from 60 to 350 ms and later bilateral frontal positivity from 200 to 300 ms in healthy participants. Both changes were specific to 1000‐Hz stimuli and were not observed for 1500 Hz, indicating stimulus specific plasticity. The latter changes are compatible with the P2 increase reported in previous research, pointing to its input specificity, while the former effect does not clearly correspond with known ERP components, thus preventing comparison with previous results.

Kompus and Westerhausen (2018) examined the effectiveness of sensory tetanization using mismatched negativity (MMN) (Näätänen et al., 2007)—the ERP component sensitive to any deviance in the sequence of repetitive auditory stimulation. MMN has been suggested as a neurophysiological index of auditory discrimination ability (Tiitinen et al., 1997) and thus is perfectly suited for such a goal. Moreover, it can be assessed in the entirely passive paradigm, where participants watch muted movies. Kompus and Westerhausen showed that after tetanization by the 13‐Hz modulation of 1025‐Hz tone, MMN specifically increased in response to this 1025‐Hz tone, but did not change in response to the neighbouring 975‐Hz tone (Kompus & Westerhausen, 2018), thus showing the input specificity of auditory tetanization. However, these authors did not examine the N1 and P2 effects in response to standard/deviant stimuli, but presented only the data on MMN differential response, leaving the question of the relationship of their results to previous findings unanswered.

The inconsistency of the tetanization effects in auditory modality and the importance of studying auditory cortex plasticity as key for language‐learning problems motivated our research. The goal of our study was to examine the neurophysiological changes provoked by the LTP‐like stimulation (tetanization) as well as its transference to the tones of neighbouring frequencies.

In addition, we aimed to probe if the tetanization effects can be observed in less challenging experimental conditions that are more appropriate, for example, for clinical groups or children. For this purpose, the experimental block that fully reproduced the original Clapp's design where participants have to fixate on the cross in the middle of the screen as used in Clapp et al. (2005), Teo et al. (2014), and Rygvold et al. (2021) was supplemented by sequences where participants watched muted movies while listening to sounds. We chose not to adopt a paradigm where participants close their eyes and listen to stimuli as used by Lei and colleagues (Lei et al., 2017), as this is prone to induction of drowsiness (Oken et al., 2006). Additionally, we implemented a high‐density EEG array with dipole localization to directly link the tetanization effect with brain regions.

## 2 MATERIALS AND METHODS

### 2.1 Participants

**FIGURE 1:**
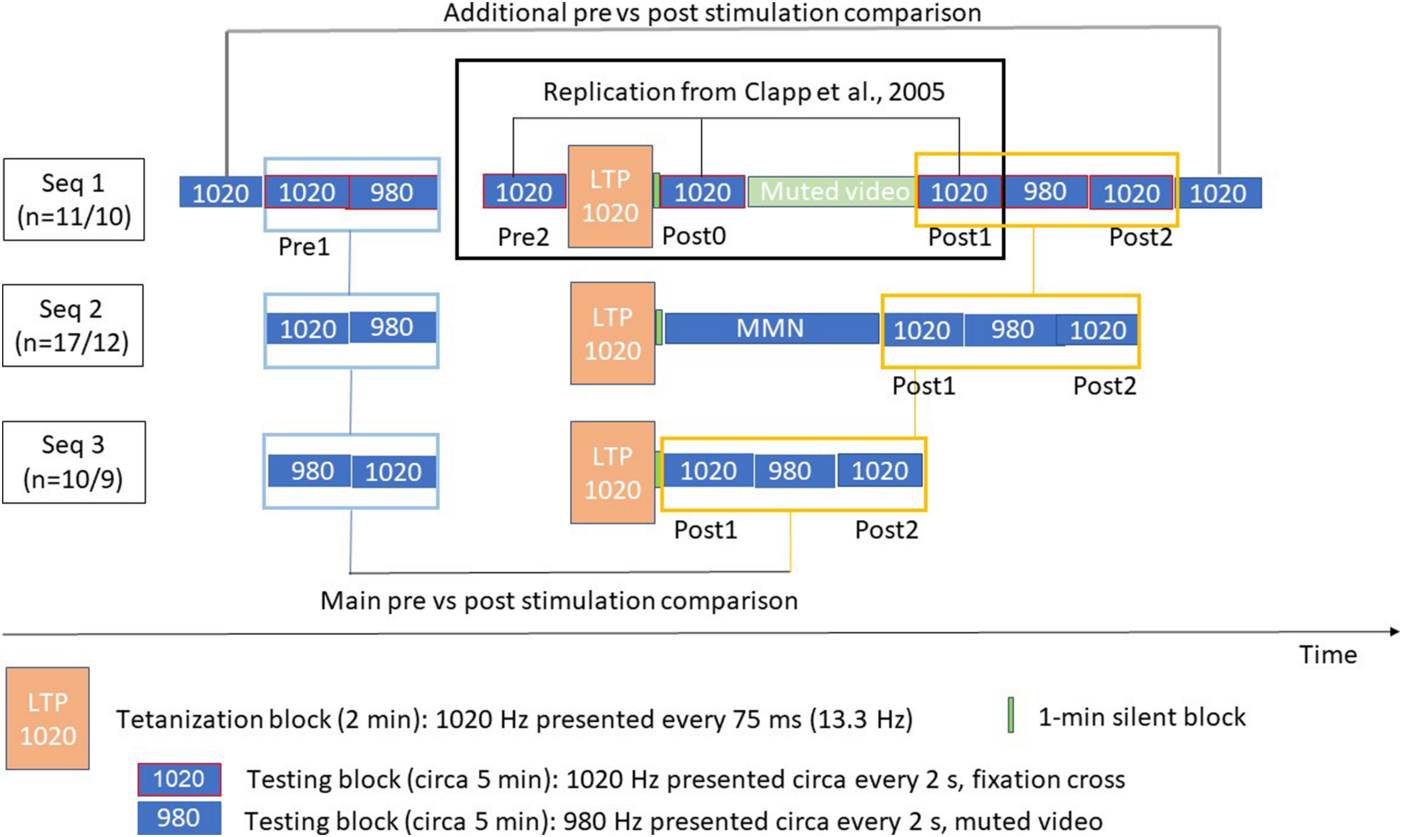
Experimental design with three experimental sequences (seq 1, seq 2, and seq 3). Filled rectangles represent different blocks: blue, those with auditory signals; green, those without auditory input. Rectangle with red border marks an active task that requires fixation on the cross, while no border corresponds with passive auditory paradigm with concurrent video watching. Orange rectangle corresponds to the tetanization block. The seq 1 contained a replication of an experimental procedure described in Clapp et al. (2005) marked with a black empty box. The additional pre‐stimulation blocks were separated from this core replication paradigm by an irrelevant supplementary task without auditory input that lasted for about 15 min. Most of the testing blocks in seq 1 require the fixation on the cross in the middle of the screen, while two blocks with passive video watching were presented in the very beginning and at the end of the sequence. Seq 2 and seq 3 included five testing blocks with a video watching (two before LTP and three after). In seq 2, the interval between stimulation and post‐stimulation (approx. 15 min) was filled with an auditory MMN oddball paradigm. One‐minute silent block separated stimulation and first post‐stimulation block in each sequence. The blue empty boxes correspond to the main pre‐stimulation testing blocks, while the yellow empty boxes to the main post stimulation blocks included in the subsequent analysis of ANOVA. *n*, number of participants in particular sequence

Thirty‐eight healthy participants (14 males, mean age 23.3 ± 5.6) took part in the study, in one of three experimental sequences (Figure 1): 11 participants (three males, mean age 24.8 ± 3.9), in the sequence 1 (seq 1); 17 participants (three males, mean age 24.6 ± 4.2), in the sequence 2 (seq 2); and 10 participants (eight males, mean age 19.5 ± 7.6), in the sequence 3 (seq 3). Due to technical issues, some experimental blocks were not recorded or excluded in several participants (one from seq 1, five from seq 2, and one from seq 3).

The participants were recruited through the advertisements at the educational events being held at the site. Before the experimental procedure, the nature of the study was explained to the participants. They were allowed to withdraw from the experiment at any time. After all the clarifications, the participants signed informed consent. At the end of the experiment, they received monetary gratification (500 rubles). The study protocol was approved by the Ethics Committee of the Institute of Higher Nervous Activity and Neurophysiology of the Russian Academy of Sciences and met the standards for research from the Helsinki Declaration of 1975.

### 2.2 Stimuli

The stimuli were represented by sinusoidal tones of two frequencies: 1020 Hz (tetanized stimulus) and 980 Hz (control stimulus). The control stimuli were added to examine the transference of tetanization effect to the tone of neighbouring frequency. The duration of each tone was 50 ms, and the loudness was at 75 dB. The interstimulus interval randomly varied from 1800 to 2600 ms. In seq 1, each tone was presented 120 times and in seq 2 and seq 3 for 150 times. LTP‐like stimulation (tetanization) lasted 2 min and consisted of the tetanized tone of 1020 Hz presented every 75 ms (roughly corresponding to 13.3‐Hz frequency). The stimuli were presented binaurally through earphones. Additional MMN block was introduced for some participants (see Section 2.3). This block consisted of a standard tone of 1000 Hz interspersed with two deviants of 1020 Hz and 980 Hz presented with the probability of 5% each. In the MMN block, the interstimulus interval was 400 ms. The results of this block will be reported elsewhere.

### 2.3 Procedure

Our experimental design included three experimental sequences to examine if the slight modification in the experimental paradigm has an impact on the tetanization effect (Figure 1). During sound presentation, participants either watched self‐selected muted movies (video) or had to look at a fixation cross in the middle of the screen. Sequence 1 (seq 1) contained a full replication of the experimental design described in Clapp et al. (2005) (blocks 5–10), as well as several additions. The exact order of blocks was as follows:

1. 1020 Hz, video;
2. 1020 Hz, fixation cross;
3. 980 Hz, fixation cross;
4. Supplementary task without auditory input, unrelated to the experiment (about 15 min);
5. 1020 Hz, cross;
6. LTP‐like stimulation/tetanization;
7. Silent period, 1 min;
8. 1020 Hz, cross;
9. Muted video, 15 min;
10. 1020 Hz, cross;
11. 980 Hz, cross;
12. 1020 Hz, cross;
13. 1020 Hz, video.

Note that additional blocks were separated from Clapp's blocks by a 15‐min supplementary task that did not contain any auditory input to prevent potential contamination of preceding auditory input with the tetanization effect. Experimental sequences 2 (seq 2) and 3 (seq 3) contained only five blocks: two before stimulation (980 Hz and 1020 Hz) and three after it (1020 Hz, 980 Hz, and 1020 Hz). The additional 1020‐Hz block was introduced to eliminate the influence of block order on the effect of stimuli type. In seq 2 and seq 3, all blocks were accompanied by concurrent muted video presentations to make the experiment more tolerable for participants. The difference between seq 2 and seq 3 was in the interval between stimulation and post‐stimulation testing blocks. In seq 2, the interval was about the same as in seq 1 from stimulation till block 10 (about 15 min), but in contrast to seq 1, it was filled with other sound stimuli (oddball MMN sequence). In seq 3, this interval was only 1 min (silent block).

### 2.4 EEG recording

EEG signals were recorded in a dimly lit soundproof room using 128‐channel caps. EEG signal was recorded continuously with 500‐Hz sampling rate and 140‐Hz lowpass filtering with actiCHamp Plus amplifiers (Brain Products). Electrode impedances were kept below 15 kOm. A reference at the FCz channel was used in the EEG acquisition and then re‐referenced at the stage of preprocessing to a common average reference.

### 2.5 EEG analysis

EEG processing was performed in MNE Python software (Gramfort et al., 2013). EEG was FIR‐filtered in the 0.1‐ to 40‐Hz range twice—once forward and once backward (“zero‐double” phase in MNE Python toolbox). Based on their spectral characteristics, noisy/flat channels were interpolated. The data were divided into epochs from 100 ms before the stimulus onset and 450 ms after it. The epochs were rejected if the peak‐to‐peak signal amplitude exceeded 350e−6 V. Eye‐movement correction was made by the means of ICA‐decomposition and rejection of components corresponding to the horizontal and to the vertical eye movements. After artefact correction, re‐referencing was performed with the use of common average reference. Before averaging, we dropped the epochs where the amplitude exceeded seven standard deviations of the mean over the whole data for each participant. The resulting minimal number of epochs was 70% from the original size of the set, thus allowing for further meaningful processing of ERPs. The epochs were averaged separately per each condition with the baseline within 100 ms before the stimulus onset.

### 2.6 Statistical analysis

Repeated‐measures analysis of variances (rmANOVA) was used to examine main effects of tetanization and its interactions with stimulus type and sequence. The data for each timeframe (0–450 ms) and electrode were entered the full omnibus ANOVA with Stimulus (tetanized vs. non‐tetanized) and Tetanization (pre vs. post stimulation) as within‐subject factors and Sequence (seq 1 vs. seq 2 vs. seq 3) as between‐subject factor. Adding the sequence as between‐participants' factor, we aim to account for the slight difference in the experimental design. We are aware that this analysis is not able to adequately assess the potential dynamics of the tetanization effect through time and stated it in Section 4.1. Note that as far as we did not observe any significant and meaningful interaction with Sequence, it was not used in the second‐level cluster‐based permutation analysis that was introduced to deal with multiple comparison problem via Fieldtrip scripts (Maris & Oostenveld, 2007; Oostenveld et al., 2011). Two main contrasts were examined: (1) effect of Tetanization (Pre vs. Post combined for tetanized and non‐tetanized stimuli, in particular, Pre 1020 plus Pre 980 vs. Post 1020 plus Post 980); (2) effect of interaction between Tetanization and Stimulus (Pre–Post differences compared for tetanized and non‐tetanized stimuli, in particular, Pre 1020 minus Post 1020 vs. Pre 980 minus Post 980). Student's *t* test for dependent variables were used in addition to ANOVA as it allows to examine the direction of the effect and is suggested in the Fieldtrip webpage for application of cluster‐based permutation test to the examination of interactions between factors. The cluster‐based permutation test was computed by randomly exchanging data between the two conditions (e.g., Pre vs. Post stimulation) and producing the maximal positive and negative cluster of each permutation (500 permutations). The effect should be observed in at least two neighbouring channels to form a cluster. Alpha level for significant clusters was set to 0.05. The significant cluster indicates the significant difference between conditions. To eliminate the contamination of the factor “block order/timing after tetanization” on the effect of stimuli type we average over Post1 and Post2 1020‐Hz blocks, appearing before and after 980 Hz, respectively. This procedure was justified by the fact that Post1 and Post2 ERPs were similar as no clusters of significant difference were formed (*p* > 0.3 supporting information). As seven participants did not have the latter 1020‐Hz block (Post2), the total *N* for ANOVA was 31. We also additionally examined the experimental sequence 1 condition using *t* test to directly replicate analysis used in Clapp et al. (2005). To estimate the effect's size, we computed Cohen's *d*.

### 2.7 Source localization

We performed inverse modelling using Brainstorm software, default anatomy, and a three‐shell spherical head model to estimate the cortical sources underlying the effects observed in sensor space. The latencies were chosen according to the ERP peaks in the components' windows determined by a cluster‐based permutation test. The activity of the sources was reconstructed using an inverse kernel obtained as a result of minimum norm imaging (current density map) with unconstrained source orientations. The noise covariance was computed from the baseline, the SNR was assigned 3, and the order of depth weighting was equal to 0.5.

## 3 RESULTS

**FIGURE 2:**
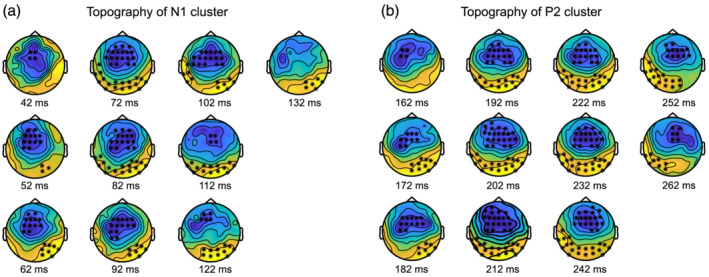
Significant clusters, obtained in the comparison of pre‐ vs. post‐ stimulation ERPs: Topography and time course. (a) Fronto‐central (negative, *p* = 0.005) and posterior (positive, *p* = 0.008) clusters for the 42‐ to 132‐ms timeframes and (b) fronto‐central (negative, *p* = 0.001) and posterior (positive, *p* = 0.001) clusters for the 162‐ to 262‐ms timeframes. “x” and “*” show the location of electrodes from the cluster

**FIGURE 3:**
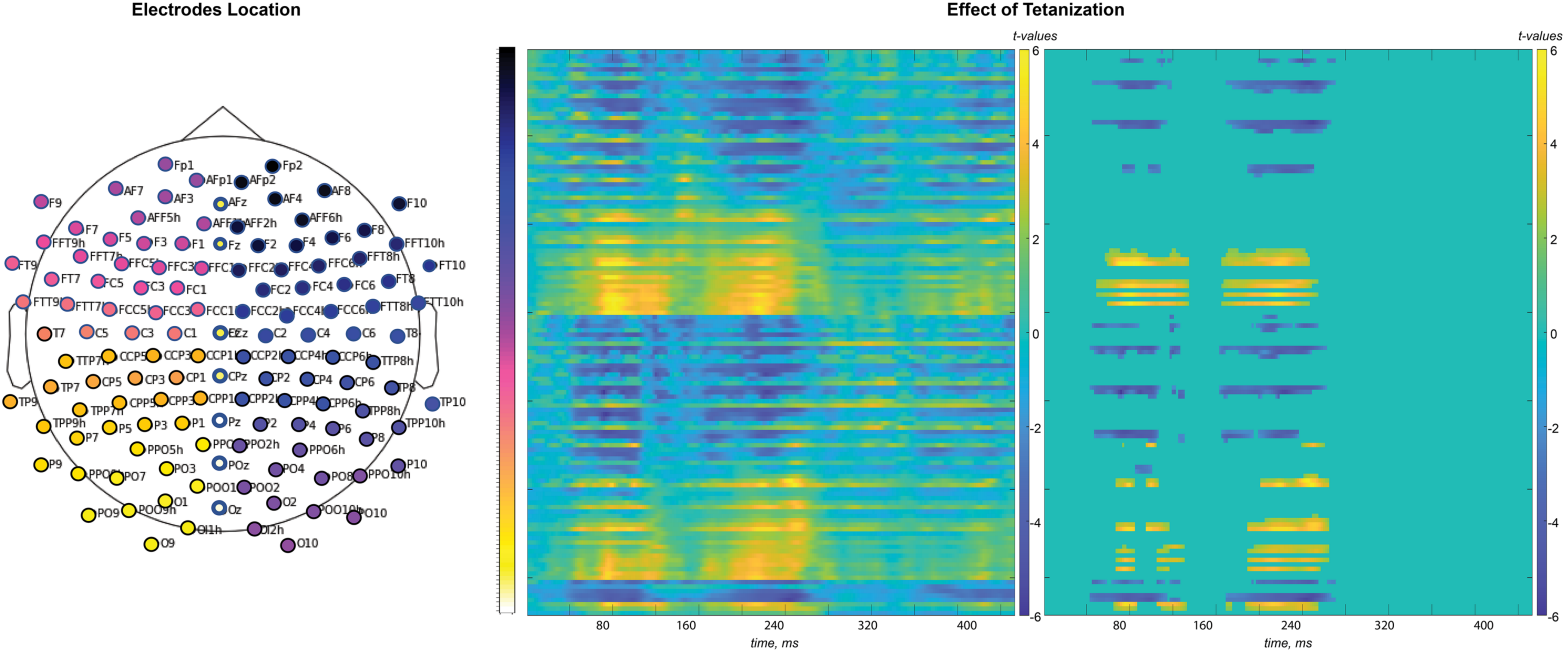
Full matrix (electrodes by timeframes) of *t* values obtained for the Tetanization effect (comparison of pre‐ and post‐tetanization ERPs). At the left panel, all *t* values are represented, while at the right, only those included into the significant clusters formed with cluster permutation test (*p* value < 0.05). Electrodes are coded by colours represented in the layout

**FIGURE 4:**
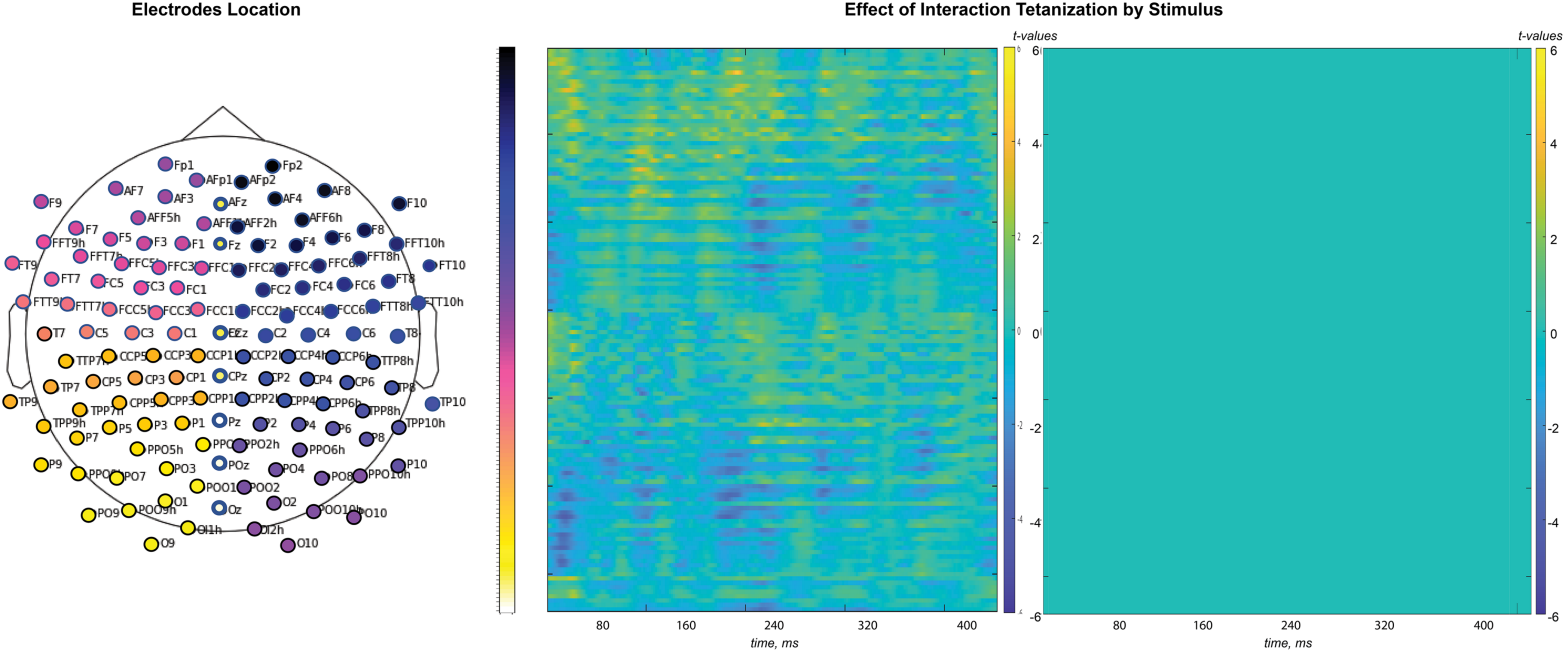
Full matrix (electrodes by timeframes) of *t* values obtained for the Tetanization by Stimulus interaction effect (comparison of pre‐ minus post‐tetanization ERPs for tetanized and not‐tetanized stimuli). At the left panel, all *t* values are represented, while at the right, only those included into the significant clusters formed with cluster permutation test (*p* value < 0.05), which is non in the current case. Electrodes are coded by colours represented in the layout

**FIGURE 5:**
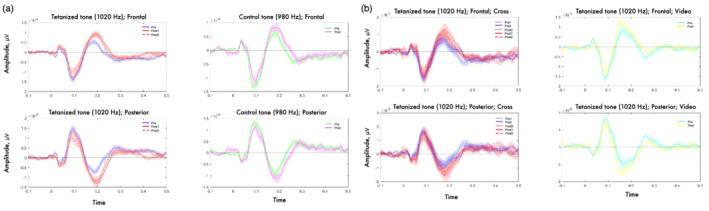
ERPs corresponding to spatiotemporal clusters revealed by cluster‐based randomization tests: (a) ERPs from all the sequences at frontal and posterior sites (grand‐averaged over the whole group, *n* = 31) for tetanized (right panel) and control (left panel) stimuli respectively; (b) ERPs at frontal and posterior sites for seq 1 (grand‐averaged over the seq 1 group, *n* = 10) for tetanized stimuli during fixation on the cross (right panel) and video presentation (left panel). Different types/colour of lines correspond with different time points of measurements in relation to tetanization. Shaded area corresponds to the interval within one SE

**FIGURE 6:**
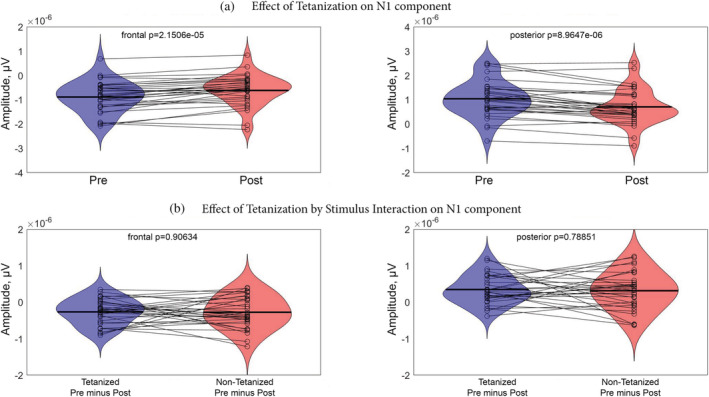
Violin plots representing distributions of ERPs amplitudes within N1 latency ranges. (a) Effect of Tetanization averaged over stimuli type for fronto‐central and posterior clusters, which is highly significant. (b) Effect of Tetanization by Stimulus interaction, which is insignificant. Dots are representing individual values, lines connect values of the same persons, obtained in different conditions

**FIGURE 7:**
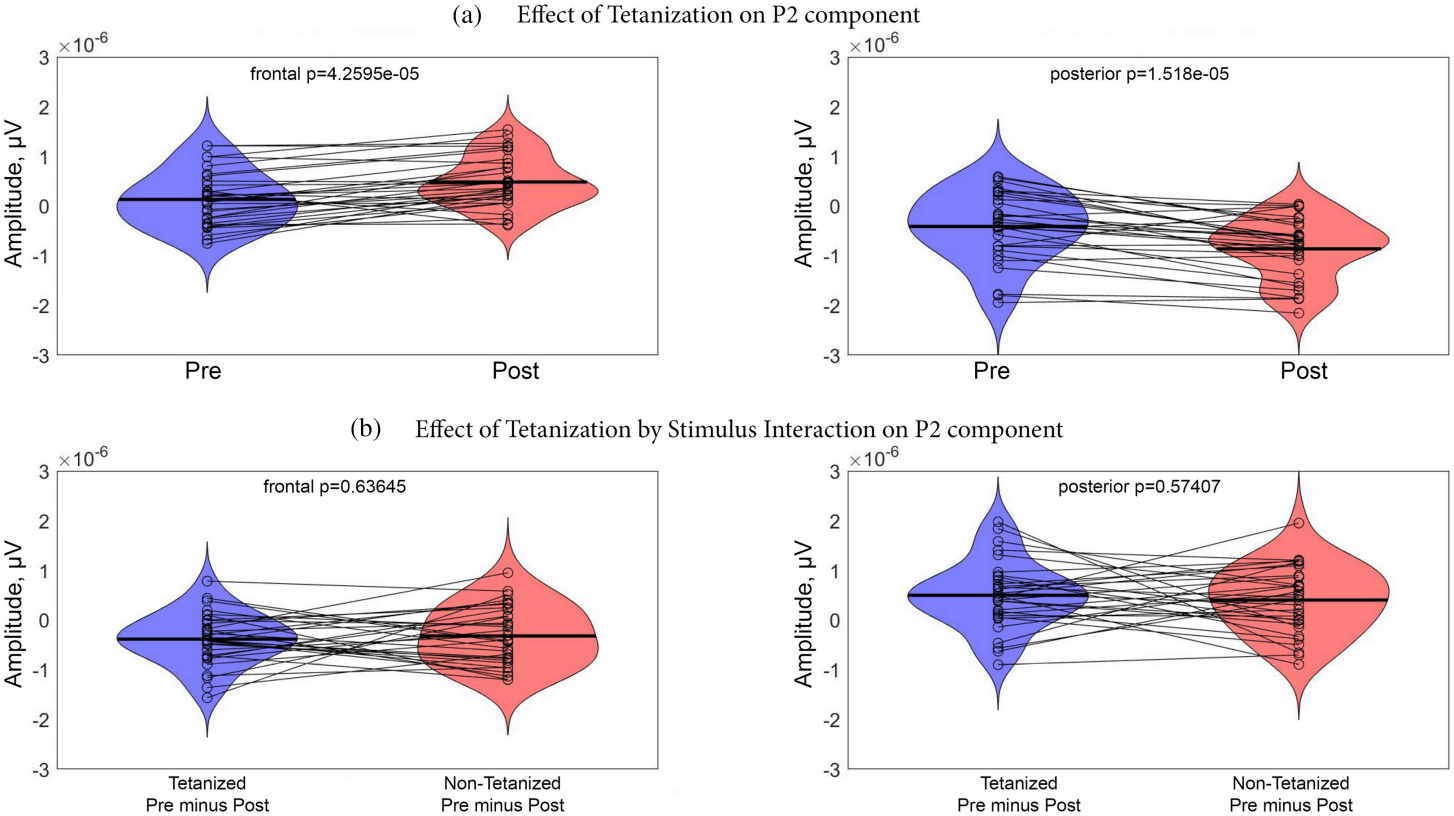
Violin plots representing distributions of ERPs amplitudes within P2 latency ranges. (a) Effect of Tetanization averaged over stimuli type for fronto‐central and posterior clusters, which is highly significant. (b) Effect of Tetanization by Stimulus interaction, which is insignificant. Dots are representing individual values, lines connect values of the same persons, obtained in different conditions

The results of rmANOVA applied to our data are summed by the full matrix (time frames by electrode) of *F* statistics that can be seen in the supporting information. No significant (*p* < 0.001) and meaningfully clustered interaction with factor Sequence was observed. The only effect that stands out was the main effect of Tetanization. To account for the multiple comparison problem, we applied cluster‐based permutation tests that confirmed significant clusters for the effect of Tetanization: pairs of positive (posterior) and negative (fronto‐central) clusters for the two periods 42–132 ms and 162–268 ms, corresponding to N1 and P2 component time windows, respectively (*p* < 0.05). The topography of the effects is represented in Figure 2 with electrodes included in the significant cluster marked by asterisks. Figure 3 represents the full electrode × time matrix for this effect. No cluster reached significance for the interaction between Stimulus and Tetanization (*p* > 0.3) as represented in Figure 4. Thus, tetanization influences similarly tetanized as well as non‐tetanized stimuli of neighbouring frequency. ERPs waveform averaged over fronto‐central and posterior sites taken from the results of cluster‐randomization analysis are shown on Figure 5. It points to the early cluster correspondence with significant attenuation of N1 components after tetanization, while the later cluster characterizes the significant post‐tetanization increase of P2 component. The ERPs for tetanized and non‐tetanized tones are plotted separately to additionally illustrate the similarity of the ERPs changes across stimuli. To show the distribution of the effects within the sample, data were averaged for the early cluster (N1) and late cluster (P2) over the fronto‐central and posterior clusters, and individual values were plotted in the Figures 6 and 7, respectively.

To compare the results with previous studies, we also examined N1 and P2 effects at common fronto‐central electrodes (N1 cluster obtained in a cluster‐randomization analysis) at peak latencies and N1‐P2 peak‐to‐peak amplitude. For this analysis, we took values around grand‐average peak amplitude (N1 80–100 ms and P2 180–200 ms). We run the same repeated‐measures ANOVA for these slightly differently calculated values. Effect of tetanization was confirmed for N1 and P2 amplitude, effect of Condition *F*(1, 28) = 15.79, *p* = 0.0004, *d* = 1.451 and *F*(1, 28) = 13.11, *p* = 0.0011, *d* = 1.322, respectively; however, no significant effects was found for N1‐P2 peak‐to‐peak amplitude, including no effect of Condition, *F*(1, 28) = 0.165, *p* = 0.688, *d* = 0.148. This result indicates that the P2 increase after tetanization might be linked to a parallel decrease in the N1, as the N1 is not fully returned to baseline at the timeframes of the P2 peak. At the same time, the effect at the later part of the P2 (e.g., 200–260 ms) is less likely to be related to N1 changes. To examine further the interdependence of tetanization effects observed at N1 and P2 latencies, we calculated the Pearson correlation between the differences in N1 and P2 obtained before and after tetanization (N1 before‐after and P2 before‐after). This correlation was not significant, *R*(30) = −0.09, *p* = 0.62, suggesting that changes of N1 and P2 after tetanization were induced by different mechanisms.

In addition, data from experimental sequence 1 were analysed separately. As you can see in Figure 5b, ERPs changes in this sequence closely resemble those observed for the whole sample (Figure 5a): N1 is decreased, while P2 is increased after the stimulation both in the blocks where participants were watching muted movies or were looking at the fixation cross in the middle of the screen during the testing. Nonetheless, *t* test analysis applied to the averaged amplitude over frontal and posterior clusters revealed the absence of any significant difference before and after the stimulation for tetanized stimuli accompanied by fixation cross—N1: *t*(10) = 1.2038, *p* = 0.25, *d* = 0.761; P2: *t*(10) = 0.0361, *p* = 0.97, *d* = 0.023—while tetanized stimuli paralleled by video presentation showed insignificant decrease in N1, *t*(10) = 1.8905, *p* = 0.087, *d* = 1.196, and significant increase in P2, *t*(10) = 3.9017, *p* = 0.003, *d* = 2.468, following LTP‐like stimulation.

**FIGURE 8:**
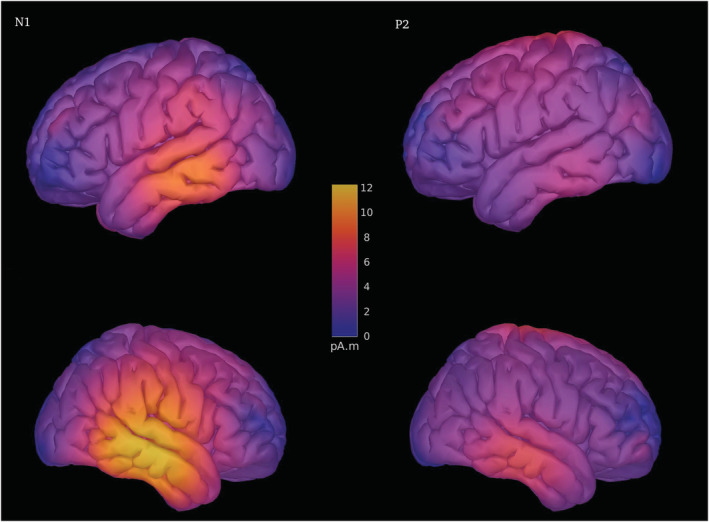
The current density distribution of N1 and P2 components reconstructed with MNE. No masking was used for visualization

The current density maps were reconstructed with MNE (see Figure 8) based on the evoked data from all the conditions to illustrate the dominant source of activity, contributing to the observed effects. This analysis demonstrates activation of temporal areas, including the primary auditory cortex and auditory association area in the superior temporal gyrus as well as the middle temporal gyrus. The coordinates of maximal activation corresponded to the right temporal lobe (for N1: *x* = 71.6, *y* = −22.0, *z* = −11.4; for P2: *x* = 70.5, *y* = −21.7, *z* = −16.3 [in millimetres] in MNI coordinates).

## 4 DISCUSSION

In this study, we investigated the ERP effects of sensory tetanization in the audition that are linked to crucial mechanisms of learning and plasticity, long‐term potentiation (LTP). Our results provide strong evidence for the changes in neurophysiological response to the auditory stimuli after tetanization, which can be potentially used in clinical research as biomarkers of LTP‐like plasticity. Unfortunately, we were not able to fully resolve the inconsistency in the results obtained in previous ERP studies: No N1 increase but decrease was observed in our study, as well as N1‐P2 peak‐to peak amplitude increase was also not replicated. Nonetheless, findings from our research are shedding light on mechanisms of sensory learning and inform a better clinical paradigm, as we discuss below.

N1 component of ERP was significantly decreased after tetanization, representing a novel finding in this type of paradigm as previous studies reported an increase (Clapp et al., 2005; Lei et al., 2017) or no changes in N1 following similar stimulation (Rygvold et al., 2021; Teo et al., 2014). As we did not replicate the Clapp and colleagues' finding even in the identical to Clapp et al. experimental condition, we believe that N1 increase might represent some uncontrolled parameters and be specific to a particular subgroup of participants as sample size in Clapp et al. (2005) study is very low (*n* = 6). Indeed, several studies pointed towards the influence of inter‐individual differences and environmental factors on LTP‐like effects (Sanders et al., 2018). Lei et al. (2017), who also reported N1 increase after tetanization, reported ERP from posterior sites, where the response can be considered as the posterior counterpart of frontal P2 component, thus not in contrast with our finding. The largest auditory tetanization study of Rygvold and colleagues (Rygvold et al., 2021, *n* = 93) while did not report a significant decrease in N1 component after tetanization in general, however, shows N1 component decrease for the conditions Post‐HFS 3 and Post‐HFS 4 (their fig. 4b). More prominent N1 decrease in our study might be related to the more optimal experimental paradigm or analysis.

While N1 decrease after 13‐Hz stimulation was not reported previously for neurotypical population, N1 attenuation after repetitive stimulation is a well‐known phenomenon in EEG research that usually refers to repetition suppression and adaptation (Briley & Krumbholz, 2013; Grill‐Spector et al., 2006; Lanting et al., 2013). These processes suggest synaptic depression or slow hyperpolarization and, thus, suppression of overall neuronal activity. The decrease of N1 in our study cannot be related to the mere effect of stimuli repetition, as we did not observe any differences in ERPs between successive blocks before stimulation. Thus, auditory tetanization is likely to cause not only potentiation but also depression.

While our study shows the depression within the N1 latencies (~100 ms) after tetanization, the later P2 component was enhanced—the finding also reported in previous research and thus representing a more reliable and consistent effect (Mears & Spencer, 2012; Rygvold et al., 2021; Teo et al., 2014). While studies of repetitive suppression often show a synchronous decrease in the N1 and P2 components (Lanting et al., 2013; Mazer et al., 2021; Sambeth et al., 2004), many studies show isolated changes in these components in different experimental approaches (de Boer & Krumbholz, 2018; Hsu et al., 2014), which supports the assumption that these components reflect rather independent neurophysiological processes (Crowley & Colrain, 2004). Our work is in line with this view as N1 and P2 showed opposite as well as uncorrelated changes after stimulation. Noteworthy, P2 increase was also reported after visual tetanization, and it was even more reliable than a more generally reported in visual tetanization studies N1b effect: Visual P2 change was present regardless of the participants' age (Spriggs et al., 2018). Several studies suggested the potentially common mechanisms underlying the P2 component across modalities, while the data are inconsistent (review in Crowley & Colrain, 2004). Nonetheless, we believe that P2, at least in the audition, is linked to consolidation processes associated with auditory memory formation and learned relevance (Tremblay et al., 2014). Several studies showed that auditory P2 is increased after perceptual learning sessions (Atienza et al., 2002; Reinke et al., 2003; Tremblay et al., 2001; Wisniewski et al., 2020) as well as in musicians (Kuriki et al., 2006; Shahin et al., 2003), suggesting its relevance to the level of expertise with sounds. Moreover, P2 increase was associated with speeded reaction time to the trained stimulus (Talebi et al., 2015; Tong et al., 2009) linking P2 enhancement to more effective task performance. Thus, we suggest that short‐term (2‐min) auditory tetanization induces neurophysiological mechanisms associated with perceptual learning and stimuli expertise that usually require several days of sessions.

There is no consistency in the questions about specificity and/or generalization of perceptual learning in the auditory domain (Fahle, 2005; Irvine, 2018). Behavioural studies reported at least partial transference of perceptual training into not‐trained stimuli (Wright & Zhang, 2009). It was proposed that early stages of processing tend to be more specific for low‐level stimulus features and reflect a high specificity of perceptual learning, whereas generalization is more compatible with a higher level of neuronal plasticity (Fahle, 2005; Karmarkar & Buonomano, 2003). Animal studies suggest that the effect of perceptual learning is partially generalizable as the frequency discrimination task leads to receptive field enlargement in the auditory cortex not only to the trained frequency but also to the neighbouring frequencies (Polley et al., 2006).

In the current study, the changes in N1 and P2 occurring after tetanization were not specific to tetanized stimuli (1020 Hz) and also occurred for the control stimulus with the frequency close to the original one (980 Hz). At the same time, when the control stimuli differed significantly from tetanized stimulus as in the study of Mears and Spencer (2012) (1000 vs. 1500 Hz) P2 increase was specific to tetanized stimuli. Specificity of the P2 increase to the trained stimuli was also reported after explicit training of stimuli discrimination in the noise for the more neighbouring stimuli (861 Hz and 1058 Hz) (Wisniewski et al., 2020). We took our stimuli from the work of Kompus and Westerhausen (2018) who reported a specific to the tetanized stimuli (1025 Hz) neurophysiological increase of mismatch negativity response (MMN) as opposed to the very neighbouring tone of 975 Hz. Unfortunately, the authors did not analyse/present N1 or P2 components only the MMN—the component calculated from a difference wave obtained by subtraction of ERPs in response to frequent standard from those to rare deviant stimuli. Thus, we cannot directly compare our results. However, considering the available evidence we can propose that the tetanization effect is different within the hierarchy of auditory information analysis. At the level of N1, which reflects the coding of physical properties of auditory stimuli, we observed a non‐specific attenuation of response. At the level of MMN, there is a specific enhancement of the pre‐attentive memory representation of stimuli within a fine‐grained tonotopic map. Furthermore, at the level of categorical representation, corresponding to the P2 component, there is a less specific amplification of the neurophysiological response within a more gross tone representation map. Noteworthy that the P2 increase in visual modality was also not specific to tetanized orientation (Spriggs et al., 2018) suggesting a potentially common cross‐modal process of post‐tetanization changes.

Most humans can perceive and resolve frequency differences between 1020 and 980 Hz (Kumar et al., 2016). Human auditory cortical fields are organized tonotopically into parallel circuits tuned to the best frequencies that can readily resolve this difference in order to sustain the perceptual upper limit (e.g., Howard et al., 1996). The major sources of the N1 and P2 components detected in our study were located within the auditory cortex, including primary and secondary areas. At the same time, the involvement of other areas cannot be excluded. It should be further studied in a separate study (preferably MEG) with the inclusion of more widely interspersed tetanized and control stimuli to examine the generalizability of the tetanization effect as well as its neurophysiological substrate more directly.

The tetanization effects in our study were observed for at least 30 min—the most prolonged interval examined, consistent with what was suggested for the effect of LTP. While the effect of experimental design modifications was not systematically addressed in our study, we did observe the ERP changes after tetanization regardless of participants' parallel activity, in particular, even in the entirely passive paradigm. The fact that neurophysiological effects were present even in the passive paradigm with concurrent video presentations that purposefully drive participants' attention from auditory stimulation to engaging video supports the viewpoint that attention is not required for LTP‐like changes after tetanization as also was shown in the fMRI experiment (Zaehle et al., 2007). We believe that this set up with muted video presentation is better suited for application in challenging populations such as patients or children. However, some inconsistencies from previous studies lessen the potential for clinical application of the observed ERP effects.

### 4.1 Limitation

The experimental design was limited as it did not include a control condition without tetanization. However, our results indirectly suggest that our findings are related to tetanization as no difference for the ERPs obtained in several sessions before tetanization was observed. Nonetheless, non‐specific N1 decrease and P2 increase after tetanization might represent some cumulative effect, so to fully examine this issue, the control blocks without tetanization should also be examined. Another limitation is that the dynamic of the tetanization effects after the stimulation was not systematically examined as other potential confounding factors were introduced (additional blocks, parallel activity). Further studies might need to address this issue more systematically. Furthermore, our block study design was not optimal to disentangle the effect of order of stimuli/time after tetanization and effect of stimuli type.

## CONFLICT OF INTEREST

None to declare.

## AUTHOR CONTRIBUTIONS

OS and AR developed the concept and design of the study. DK, AR, GS, DGK, and AN did the participants' recruiting and data collection. DK and GS performed the data analyses. DK and OS conducted the statistical analyses. OS, DK, and AR wrote the manuscript, and all authors contributed to and have approved the final manuscript.

### PEER REVIEW

The peer review history for this article is available at https://publons.com/publon/10.1111/ejn.15513.

## Supporting information


**Figure S1:** Full matrix (electrodes by timeframes) of F‐values obtained for the main effect of Tetanization (Pre vs Post). At the left panel all F‐values are represented, while at the right – only those with p‐value < 0.001. Electrodes are coded be colors represented in the layout. You can clearly see the significant differences around N1and P2 timeframes at multiple electrodes.

**Figure S2:** Full matrix (electrodes by timeframes) of F‐values obtained for the effect of Tetanization by Sequence interaction. At the left panel all F‐values are represented, while at the right – only those with p‐value < 0.001. Electrodes are coded be colors represented in the layout.

**Figure S3:** Full matrix (electrodes by timeframes) of F‐values obtained for the main effect of Stimulus Type (1020 Hz vs 980 Hz). At the left panel all F‐values are represented, while at the right – only those with p‐value < 0.001. Electrodes are coded be colors represented in the layout.

**Figure S4:** Full matrix (electrodes by timeframes) of F‐values obtained for the effect of Tetanization by Stimulus Type interaction. At the left panel all F‐values are represented, while at the right – only those with p‐value < 0.001. Electrodes are coded be colors represented in the layout. As only few separate points reached significance, we can conclude that effect of Tetanization is similar for both stimuli.

**Figure S5:** Full matrix (electrodes by timeframes) of F‐values obtained for the effect of Stimulus Type by Sequence interaction. At the left panel all F‐values are represented, while at the right – only those with p‐value < 0.001. Electrodes are coded be colors represented in the layout.

**Figure S6:** Full matrix (electrodes by timeframes) of F‐values obtained for the effect of Tetanization by Stimulus Type by Sequence interaction. At the left panel all F‐values are represented, while at the right – only those with p‐value < 0.001. Electrodes are coded be colors represented in the layout.

**Figure S7:** Full matrix (electrodes by timeframes) of F‐values obtained for the main effect of Sequence. At the left panel all F‐values are represented, while at the right – only those with p‐value < 0.001. Electrodes are coded be colors represented in the layout.

**Data S1.** ERP corresponding to each contrast that was tested in the cluster analysis.

## Data Availability

The data that support the findings of this study are available from the corresponding author upon reasonable request.
