# Supplementary figures and images for: Generalization of sustained neurophysiological effects of short‐term auditory 13‐Hz stimulation to neighbouring frequency representation in humans
Source: Eur J Neurosci. 2021 Dec 16;55(1):175–88. doi: 10.1111/ejn.15513 (PMC9299826; doi:10.1111/ejn.15513)

# EFFECT OF TETANIZATION

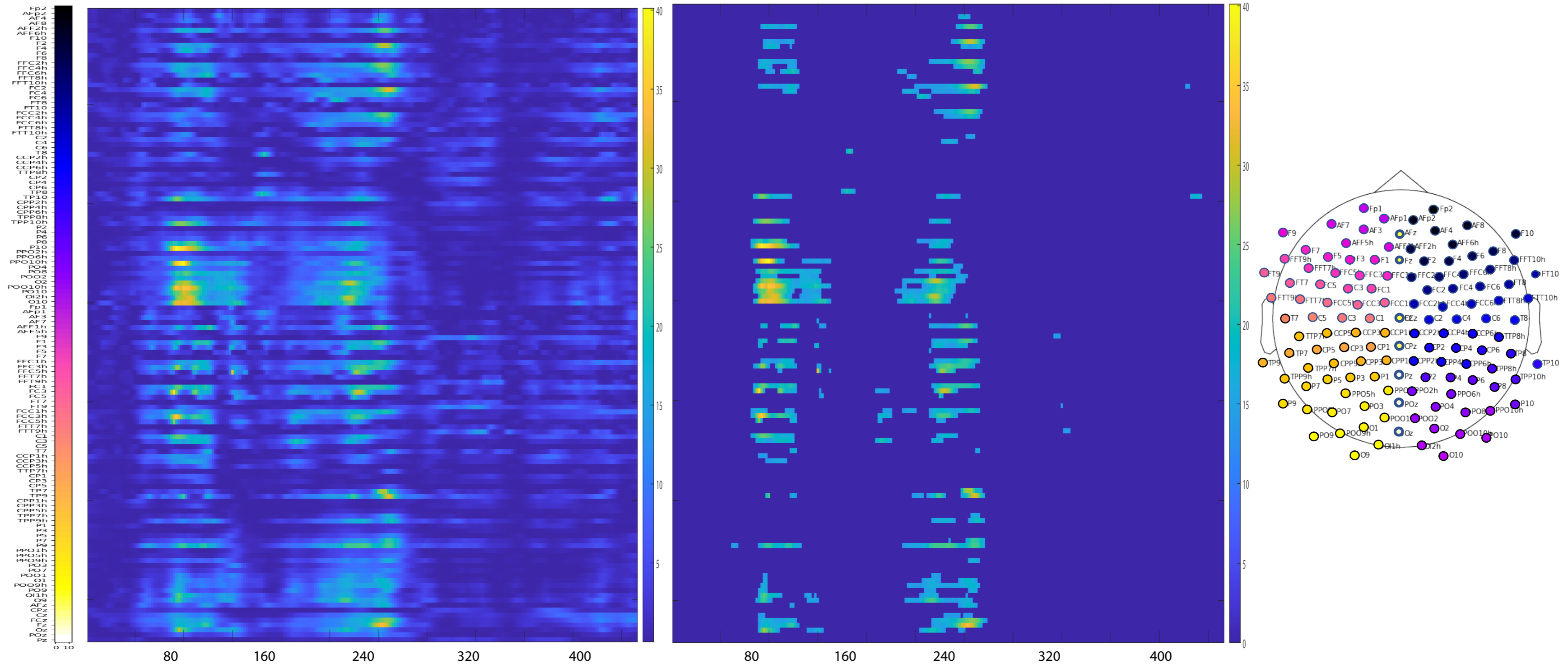

Supplement: Supplementary file 1 — Figure S1: Full matrix (electrodes by timeframes) of F‐values obtained for the main effect of Tetanization (Pre vs Post). At the left panel all F‐values are represented, while at the right – only those with p‐value < 0.001. Electrodes are coded be colors represented in the layout. You can clearly see the significant differences around N1and P2 timeframes at multiple electrodes. [file EJN-55-175-s003.pdf]

# EFFECT OF INTERACTION TETANIZATION BY SEQUENCE

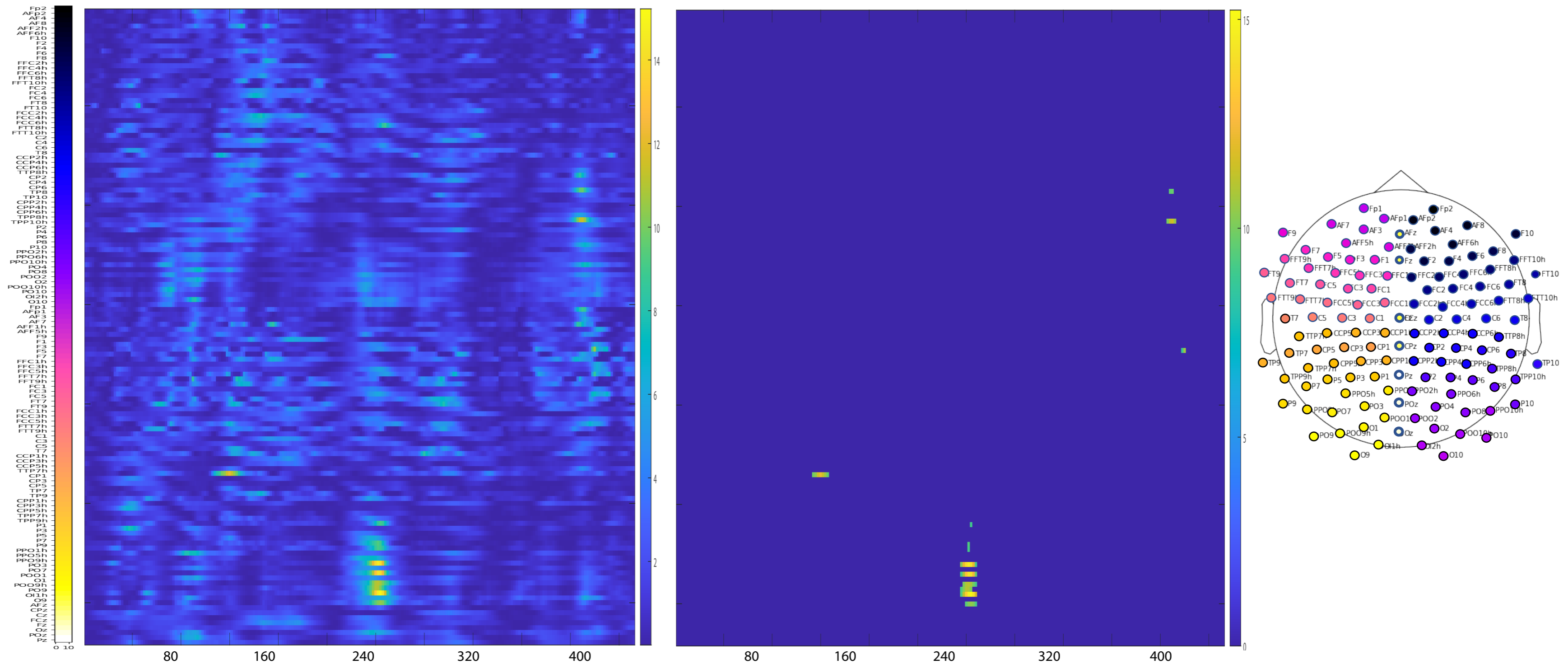

Supplement: Supplementary file 2 — Figure S2: Full matrix (electrodes by timeframes) of F‐values obtained for the effect of Tetanization by Sequence interaction. At the left panel all F‐values are represented, while at the right – only those with p‐value < 0.001. Electrodes are coded be colors represented in the layout. [file EJN-55-175-s006.pdf]

# EFFECT OF STIMULUS TYPE

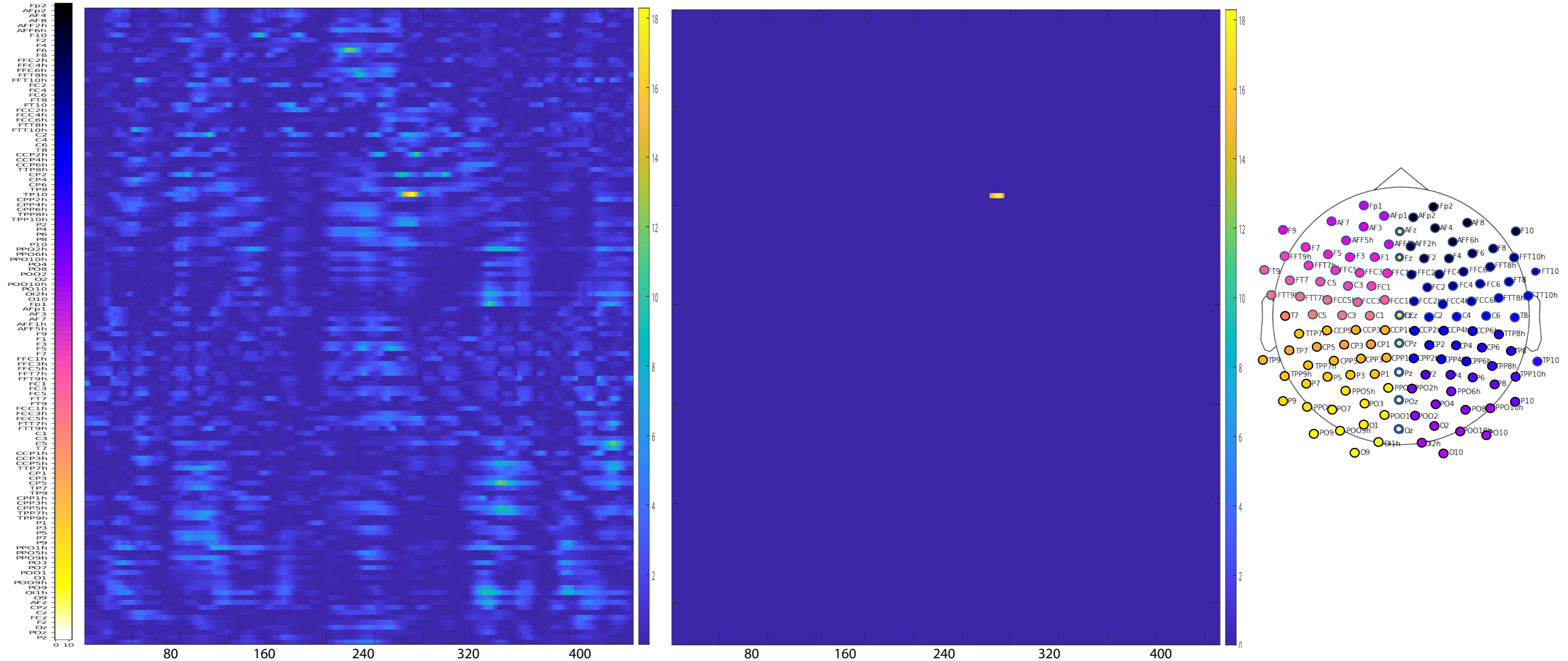

Supplement: Supplementary file 3 — Figure S3: Full matrix (electrodes by timeframes) of F‐values obtained for the main effect of Stimulus Type (1020 Hz vs 980 Hz). At the left panel all F‐values are represented, while at the right – only those with p‐value < 0.001. Electrodes are coded be colors represented in the layout. [file EJN-55-175-s008.pdf]

# EFFECT OF INTERACTION TETANIZATION BY STIMULUS TYPE BY SEQUENCE

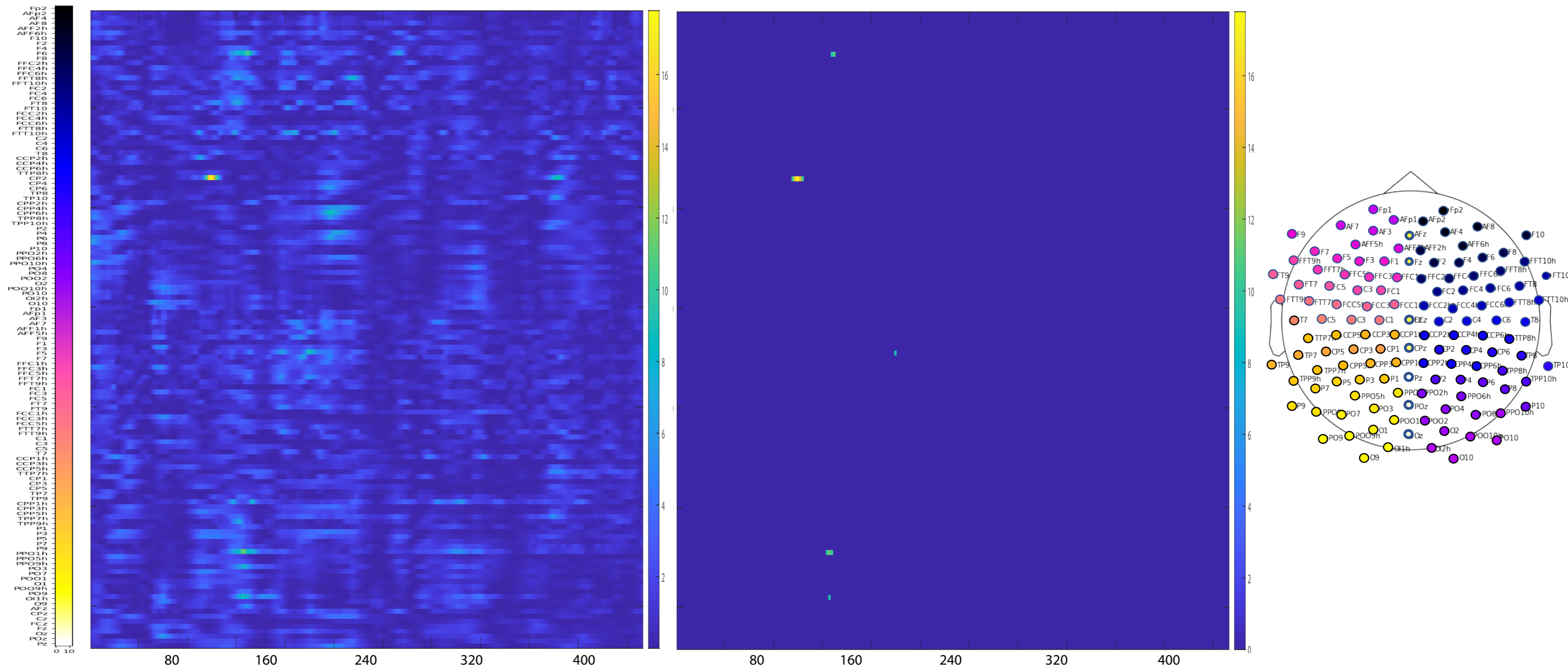

Supplement: Supplementary file 6 — Figure S6: Full matrix (electrodes by timeframes) of F‐values obtained for the effect of Tetanization by Stimulus Type by Sequence interaction. At the left panel all F‐values are represented, while at the right – only those with p‐value < 0.001. Electrodes are coded be colors represented in the layout. [file EJN-55-175-s004.pdf]

# MAIN EFFECT OF SEQUENCE

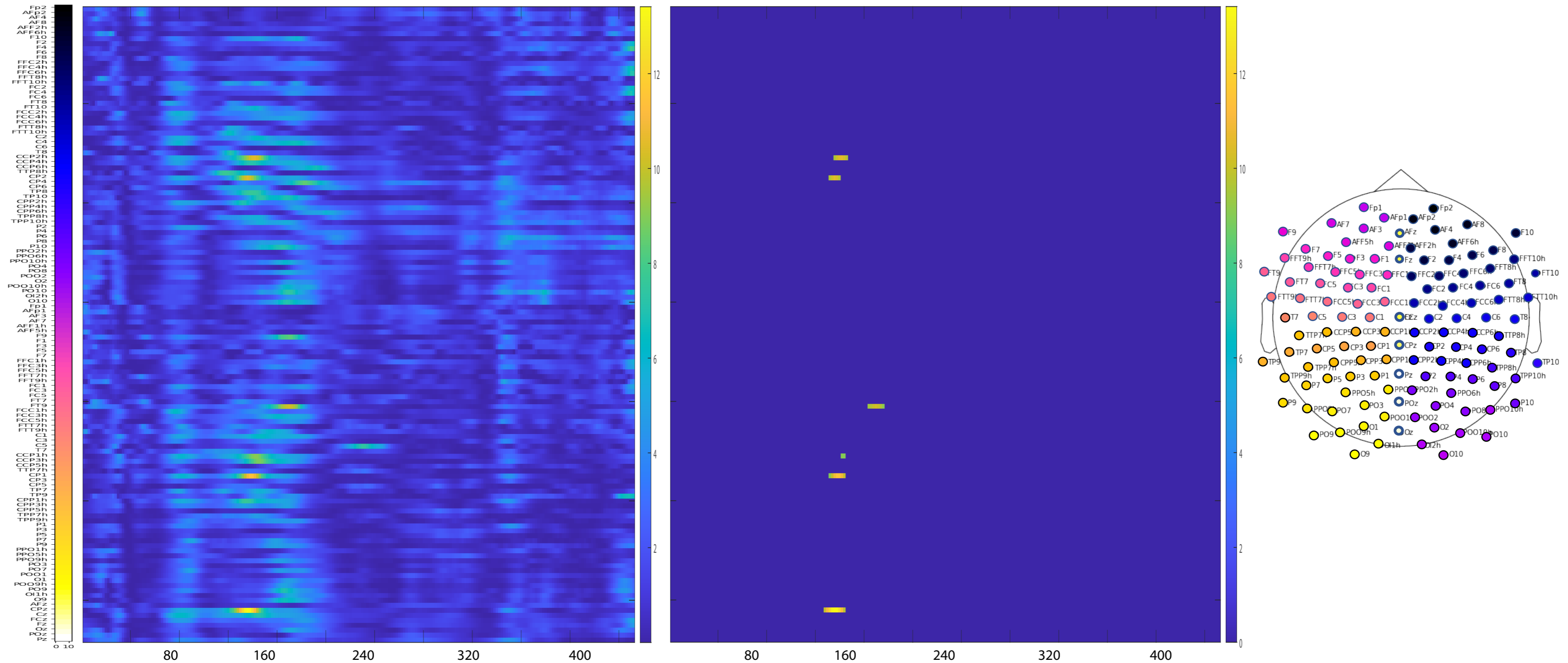

Supplement: Supplementary file 7 — Figure S7: Full matrix (electrodes by timeframes) of F‐values obtained for the main effect of Sequence. At the left panel all F‐values are represented, while at the right – only those with p‐value < 0.001. Electrodes are coded be colors represented in the layout. [file EJN-55-175-s002.pdf]
