# Supplementary material for: Generalization of sustained neurophysiological effects of short‐term auditory 13‐Hz stimulation to neighbouring frequency representation in humans
Source: Eur J Neurosci. 2021 Dec 16;55(1):175–88. doi: 10.1111/ejn.15513 (PMC9299826; doi:10.1111/ejn.15513)

Supplementary figure 1. ERP corresponding to each contrast that was tested in the cluster analysis.


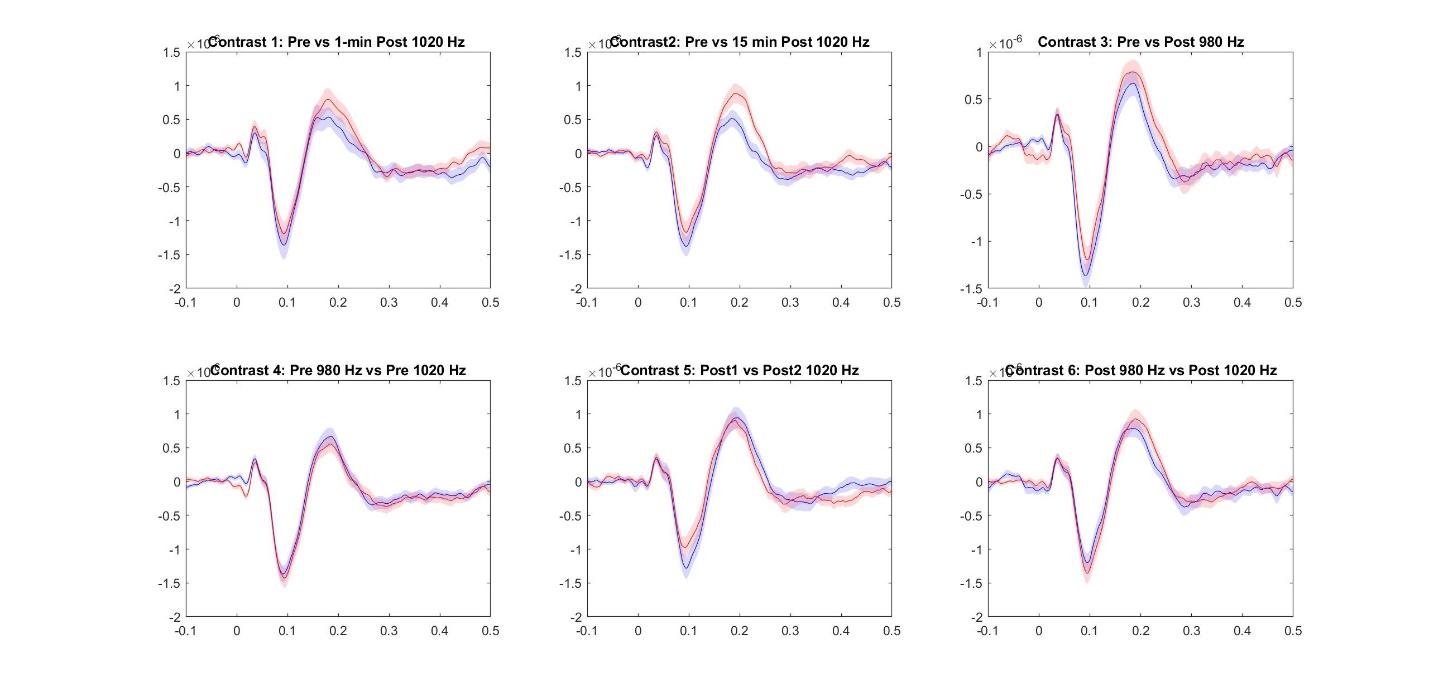

Supplement: Supplementary file 8 — Data S1. ERP corresponding to each contrast that was tested in the cluster analysis. [file EJN-55-175-s005.docx]
