# Supplementary material for: Generalization of sustained neurophysiological effects of short‐term auditory 13‐Hz stimulation to neighbouring frequency representation in humans
Source: Eur J Neurosci. 2021 Dec 16;55(1):175–88. doi: 10.1111/ejn.15513 (PMC9299826; doi:10.1111/ejn.15513)

EFFECT OF INTERACTION TETANIZATION BY STIMULUS TYPE

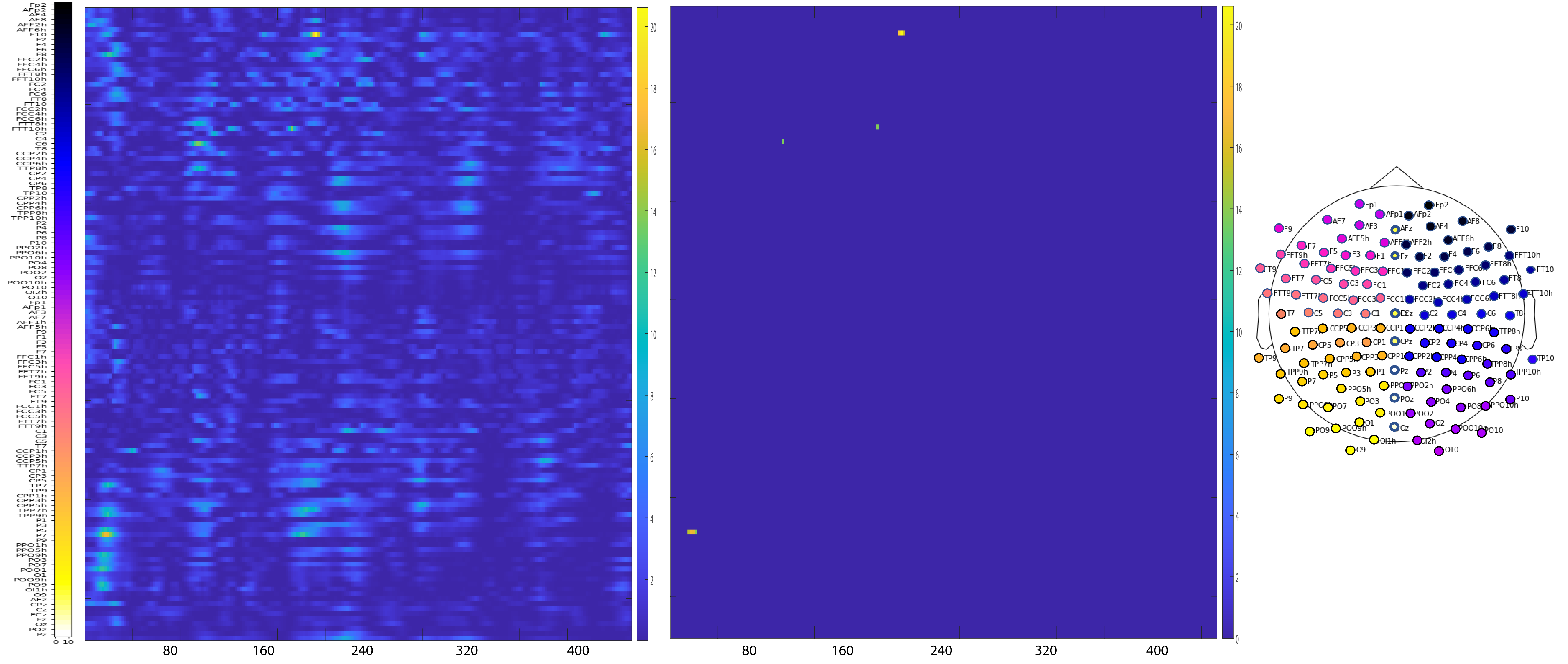

Supplement: Supplementary file 4 — Figure S4: Full matrix (electrodes by timeframes) of F‐values obtained for the effect of Tetanization by Stimulus Type interaction. At the left panel all F‐values are represented, while at the right – only those with p‐value < 0.001. Electrodes are coded be colors represented in the layout. As only few separate points reached significance, we can conclude that effect of Tetanization is similar for both stimuli. [file EJN-55-175-s001.pdf]
