# Supplementary material for: Generalization of sustained neurophysiological effects of short‐term auditory 13‐Hz stimulation to neighbouring frequency representation in humans
Source: Eur J Neurosci. 2021 Dec 16;55(1):175–88. doi: 10.1111/ejn.15513 (PMC9299826; doi:10.1111/ejn.15513)

EFFECT OF INTERACTION STIMULUS TYPE BY SEQUENCE

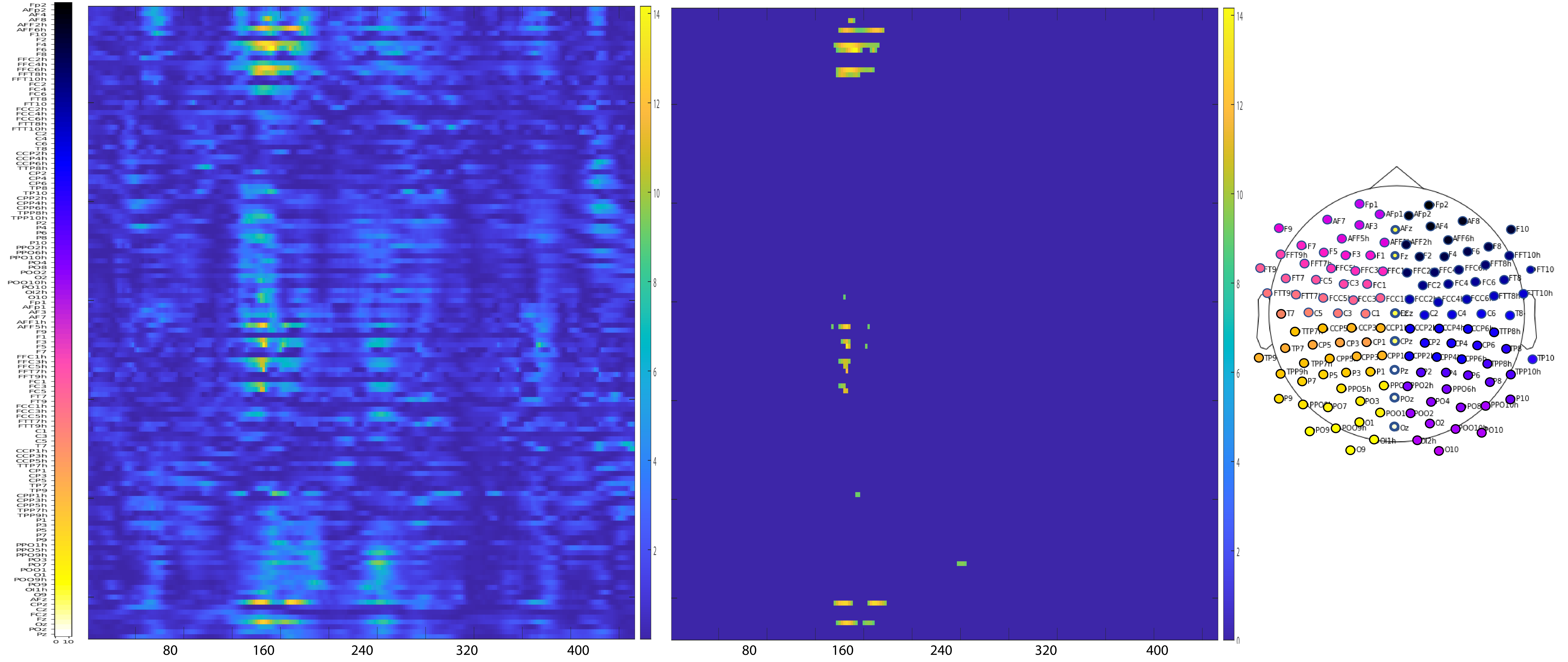

Supplement: Supplementary file 5 — Figure S5: Full matrix (electrodes by timeframes) of F‐values obtained for the effect of Stimulus Type by Sequence interaction. At the left panel all F‐values are represented, while at the right – only those with p‐value < 0.001. Electrodes are coded be colors represented in the layout. [file EJN-55-175-s007.pdf]
